# Phosphorus speciation in a prairie soil amended with MBM and DDG ash: Sequential chemical extraction and synchrotron-based XANES spectroscopy investigations

**DOI:** 10.1038/s41598-018-21935-4

**Published:** 2018-02-26

**Authors:** Khaled D. Alotaibi, Jeff. J. Schoenau, Gourango Kar, Derek Peak, Terry Fonstad

**Affiliations:** 1 Department of Soil Science, King Saud University, Box 2460, Riyadh, 11451 Saudi Arabia; 2 Department of Soil Science, University of Saskatchewan, 51 Campus Drive, Saskatoon, Sk S7N 5A8 Canada; 3 Department of Chemical and Biological Engineering, University of Saskatchewan, 51 Campus Drive, Saskatoon, Sk S7N 5A9 Canada

## Abstract

Sequential chemical extraction and synchrotron-based XANES spectroscopy techniques were used to identify P species in two ashes before and after addition to a prairie soil. The used ashes were: meat and bone meal ash (MBMA) and dried distillers grains ash (DDGA) plus mineral P fertilizer (MP) for comparison. Soil treated with MP contained higher content of resin-Pi and NaHCO_3_-Pi followed by DDGA and MBMA. The MBMA amended soil had the highest (47%) proportion of the soil P contained in recalcitrant HCl extractable fraction, reflecting more Ca-bound P present and being formed in soil after application. Analysis of both ashes with XANES spectroscopy before application to soil revealed that MBMA had strong spectral features consistent with hydroxyapatite (Ca_5_(PO_4_)_3_(OH)). DDGA exhibited spectral features consistent with a mixture of several Mg and K phosphate salts rather than a single mineral species. The distinctive features in the XANES spectra of both ashes largely disappeared after amendment to the soil, suggesting transformation to different P forms in the soil after application. It is also possible that the added amount of P to the studied soil via DDGS or MBMA was small enough so that P speciation is not different from the background P level.

## Introduction

Phosphorus is an essential element for plants, and its supply in soil is critical in maximizing crop production and improving food and feed quality. The global demand for phosphorus fertilizer is projected to increase by 50–100% by 2050 to enable sustained food production for a growing world population^1^. Complete reliance on phosphorus fertilizer manufactured from mined phosphate rock may not be a good strategy for sustained crop yields, stimulating the need to find alternative phosphorus sources and recycle more effectively the P that is contained in agricultural by-products. One option is the utilization of ash generated from organic materials via the gasification process, a thermal breakdown of organic materials under high temperature (800–900 °C) in presence of oxygen^2^. Application of this combustion technology to meat and bone meal (MBM) and dried distillers grains (DDG) as a means of providing additional nutrient and energy recovery has gained interest^3–5^. The gasification of MBM and DDG produces ash byproduct that was noted to have high content of total P, and with potential as P fertilizer source^6^. However, the P forms in the ash and ash amended soil were not examined in this study.

In general, ashes derived from various feedstocks, such as poultry litter, turkey manure and crop residues have a positive impact on crop yield and soil properties when added to soil^7–9^. However, the effects on P availability and mobility will depend on the P forms produced in the amended soil, and may vary depending on feedstock and gasification conditions. Furthermore, P in ash may exist in different forms and availability levels, and when added to soil, it is anticipated to interact/associate with soil constituents and reside in different forms as well. Therefore, knowledge of P fractions in ash and in soil after application is paramount.

A variety of approaches have been used to reveal P speciation in waste amended soil. Examples of these methods include sequential chemical extraction and X-ray absorption near-edge structure analysis (XANES). The sequential chemical extraction technique based on Hedley *et al*.^10^ has widely been applied to characterize different forms of soil P^11–16^. This technique utilizes different chemical extractants of increasing strength to separate P in soils into various chemical fractions of different bioavailability such as plant-available, Ca-associated, Al oxide and Fe oxide-associated P.

Synchrotron-based XANES spectroscopy is useful as a solid-state speciation tool to directly reveal P forms in organic amendments such as poultry litter, manures or biosolids or in soil amended with fertilizers^17–25^. The technique enables *in-situ* identification, absent of the artifacts inherent in chemical separation methods. In addition, a combination of this technique with chemical fractionation has been successfully employed in few studies to better understand P species in organic amendments or in soil amended with organic materials^18,20,21^.

The objective of the current study was to determine the nature of P in MBM and DDG ash, and in a typical prairie agricultural soil amended with these materials, using sequential chemical extraction and XANES spectroscopy. Soluble mineral fertilizer (monocalcium phosphate) was included for comparison. This study was conducted as follow-up on initial studies of plant growth responses to MBM and DDG ash reported in Alotaibi *et al*.^26^.

## Materials and Methods

### Production of ash from gasification of MBM and DDG, procurement and preparation

The ashes used in the current study were byproducts generated from gasification of two organic materials: (1) meat and bone meal and (2) dried distillers grain that were evaluated locally for their feasibility as feedstocks for biogas production. The MBM ash (MBMA) was obtained from bovine MBM cracklings that were provided by Saskatoon Processing Ltd., Saskatoon, SK, Canada. The DDG ash (DDGA) was generated from gasification of DDG provided by a wheat-based ethanol production facility near Lanigan, SK, Canada. Both products (MBM, DDG) were first ground in the lab and then fed to a two-stage fixed bed reactor, followed by a gasification process using a fluidized bed gasification pilot system developed by the Fluidization Laboratory of Saskatchewan (FLASK^TM^) at the University of Saskatchewan, Department of Chemical Engineering to produce syngas from biomasses and other carbonaceous materials^27^. Gasification of both materials occurred at atmospheric pressure and a temperature of 650–850 °C. The gasification process is described in detail by Campbell *et al*.^27^. The gasified MBM and DDG ashes were collected and ground to pass through a 600 µm sieve to obtain a homogeneous product. The resulting ash was then stored in the lab until its use. Prior to the experiment, a representative sample of each ash type was collected and analyzed for chemical composition. Basic characteristics of both ashes are provided in Table 1.

**Table 1:** Elemental analysis of meat & bone meal ash (MBMA) and dried distillers grain ash (DDGA), expressed on a dry weight basis (previously reported by Alotaibi *et al*.^26^).

Parameter	Ash type	
	MBMA	DDGA
Total C (%)	0.09	0.87
Total N (%)	0.235	0.135
Total P (%)	17.65	18.65
Total K (%)	2.88	14.9
Total S (%)	0.425	<1.0
Total Na (%)	6.55	7.45
Total Ca (%)	24.65	7.85
Total Mg (%)	1.07	5.4
Total Cu (mg g^−1^)	0.08	0.25
Total Fe (%)	0.358	0.62
Total Mn (mg g^−1^)	0.09	1.68
Total Zn (mg g^−1^)	0.69	1.19
Moisture (%)	<0.10	<0.10

### Soil collection and analysis

A large bulk soil sample was manually collected from the A horizon surface layer (0–20 cm) of a cultivated field (cereal-legume-oilseed rotation) in south-central Saskatchewan, Canada. The soil was classified as an Orthic Brown Chernozem (Aridic Kastanozem, FAO system). The bulk soil sample collected was shipped to the soil processing facility at the University of Saskatchewan (Saskatoon, SK, Canada) and mechanically mixed using a stationary mixer. A subsample of the soil was air-dried and ground to pass a 2-mm sieve and analyzed for selected properties (Table 2). Soil content of organic C was determined using a LECO CR-12 combustion carbon analyzer (LECO Corporation, St, Joseph, MI) set at 840 °C^28^. Exchangeable NH_4_^+^-N and NO_3_^–^-N were determined according the method described by Keeney and Nelson (1982)^29^. A modified Kelowna method was used to determine available P and K^30^. Soil pH and EC were measured in 1:1 soil:water suspension. Particle-size distribution was determined using pipette method^31^.

**Table 2:** Selected properties of the study soil.

Property	Value
OC (mg g^−1^)	19.00
NH_4_^+^-N (µg g^−1^)	6.1
NO_3_^–^-N (µg g^−1^)	9.7
Avail. P (µg g^−1^)	7.3
Avail. K (µg g^−1^)	450
EC (dS m^−1^)	0.19
pH	7.2
Sand (%)	52
Silt (%)	25
Clay (%)	23

### Incubation experiment set-up and treatment application

Homogenized field-moist soil (1000 g) was placed into 1-L cylindrical plastic pots and was treated with ash fertilizer. Details on experimental design and treatment application are provided in Alotaibi *et al*.^26^. Briefly, the experimental treatments included three P sources: (1) soluble mineral P fertilizer as granular mono-calcium phosphate (MP), (2) meat and bone meal ash (MBMA), and (3) dried distillers grains ash (DDGA) applied at three rates equivalent to 25, 50 and 100 kg P ha^−1^, referred to as low (L), medium (M), and high (H) rate, respectively. The rate of MBMA and DDGA application was based on their total P content. A control treatment that received no P was included. Each treatment received 200 kg N ha^−1^ as granular urea, including the control, to supply enough N to eliminate N deficiency. Moreover, as the soil used in this experiment showed sulfur deficiency and the sulfur requirement of the canola grown on the soils is high^32^, each treatment was supplied with a basal application of 40 kg S ha^−1^ as K_2_SO_4_ solution, including the control. Each treatment was replicated four times in a completely randomized design. Each pot was seeded to Argentine canola (*Brassica napus* L.L. 5030) and placed in a growth chamber that was set at 22 °C day and 13 °C night, with an 18-h day length and 6-h night length for a period of five weeks. More details about plant growth monitoring, harvesting and data collection are provided in Alotaibi *et al*.^26^. After canola plant harvest, soils were removed from the pots, air-dried and ground to pass a 2-mm sieve prior to laboratory analysis.

### Sequential chemical extraction of P

The phosphorus sequential extraction protocol used is based on Hedley *et al*.^10^ procedure as described by Tiessen and Moir (2008)^33^. In this method, a 0.5 g sample of air-dried and sieved soil was weighed into a 50-mL centrifuge tube followed by addition of 30 mL of deionized water and 2 strips of anion-exchange resin membrane. The contents were shaken for 16 h on a rotary shaker, and then the resin strips were transferred to clean 50-mL tube and shaken with 20 mL of 0.5 M HCl for 16 h to elute the P, followed by a determination of inorganic P (P_i_) as described below. The tubes containing soil suspension were then centrifuged at 10,000 × g for 10 min at 0 °C, and the liquid was discarded. Then, 30 mL of 0.5 M NaHCO_3_ (pH 8.5) was added to tubes, shaken and centrifuged as above and the supernatant was filtered through a 0.45-µm filter. The inorganic and total P (P_t_) in NaHCO_3_ extract was then determined as described below. The extraction process was repeated as above with the extractants of 0.1 M NaOH and 1 M HCl, respectively, and P_t_ and P_i_ in NaOH extract, and P_i_ in the HCl extract were determined as described below. The remaining soil residue (residual fraction) was then transferred to a 75-mL digestion tube using distilled water, and digested using concentrated H_2_SO_4_ and 30% H_2_O_2_ following the method of Thomas *et al*.^34^.

Inorganic P recovered from the resin strip and P_i_ in HCl extracts was determined directly using the method of Murphy and Riley^35^. For the NaHCO_3_ and NaOH extracts, a suitable aliquot of each extract was acidified first by adding 0.9 M H_2_SO_4_ to precipitate organic matter prior to P_i_ determination using Murphy and Riley method (1962)^35^. Total extractable P was determined in NaHCO_3_ and NaOH extracts by oxidizing the dissolved organic matter with ammonium persulfate as described by Tiessen and Moir (2008)^33^, and then the P was determined colorimetrically using Murphy and Riley method (1962)^35^. Absorbance was measured using Beckman DU-65 spectrophotometer at a wavelength of 712 nm. Inorganic P was subtracted from total P to determine organic P (P_o_) in the respective extract.

The forms of P recovered from the various extractants employed here are interpreted according to the general understanding of the action of individual extractants, their sequence, and their relationship to soil P chemical forms and conditions^33^. Resin-P is defined as labile inorganic P that is readily exchangeable and bioavailable. The NaHCO_3_-P is the labile P_i_ and P_o_ that is sorbed to soil minerals surfaces in addition to a small portion of microbial P. The NaOH-P represents P_i_ and P_o_ that are strongly chemisorbed to aluminum- and iron-oxide minerals. The HCl-P_i_ is considered to be predominantly insoluble calcium phosphate (e.g. apatite) type minerals (Ca-bound P_i_). The acid-digested P is the highly insoluble P_i_ and recalcitrant and stable P_o_.

### Phosphorus K-edge XANES spectroscopy analysis

Solid-state characterization of P from the soil samples, ash fertilizers, and P reference compounds was conducted at the Soft X-ray micro-characterization beamline (SXRMB) at the Canadian Light Source (CLS), Saskatoon, Canada. Measurements were performed with InSb(III) monochromator with focused beam size 300 µm × 300 µm. Beamline was calibrated using ZnPO_4_ powder to the edge energy of 2158 eV in total electron yield (TEY) mode spectrum. The samples were mounted on a stainless-steel sample holder using double-sided carbon tape and placed in the vacuum chamber. The P standards spectra were collected in TEY mode to minimize artifacts caused by self-absorption^25,36,37^. Soil and fertilizer sample spectra were collected in partial fluorescence yield (PFY) mode from 2120–2190 eV with 0.25 eV resolution and multiple co-added spectra were averaged to obtain adequate signal to noise ratio for analysis. All XANES spectra were analyzed using the Athena software package^38^. The averaged XANES spectra were background corrected by a first-order polynomial fit through the pre-edge region (2135–2145 eV). This was followed by normalization to an edge jump of 1.0 between 2140 and 2180 eV to facilitate comparison with samples of different P concentration.

### Statistical analysis

The experiment consisted of three amendment types (factor) with three rates of application in addition to a control. Thus, it was a completely randomized design with a complete factorial arrangement. Prior to statistical analysis, the data were checked for homogeneity using Shapiro-Wilk test. This showed that data were homogenous, and accordingly statistical analysis was conducted on the raw data. Two-way ANOVA procedure was carried out to reveal the effects of P sources (MP, MBMA, and DDGA), rate (low, medium, and high), and their interaction on P forms recovered by each extractant. Treatment effects were deemed significant at *P* < 0.05 and they were considered a trend at 0.05 < *P* < 0.10. Treatment means were separated at *P* ≤ 0.05 using Student-Newman-Keuls (SNK) test.

## Results and Discussion

### Sequential chemical analysis of P

**Figure 1:**
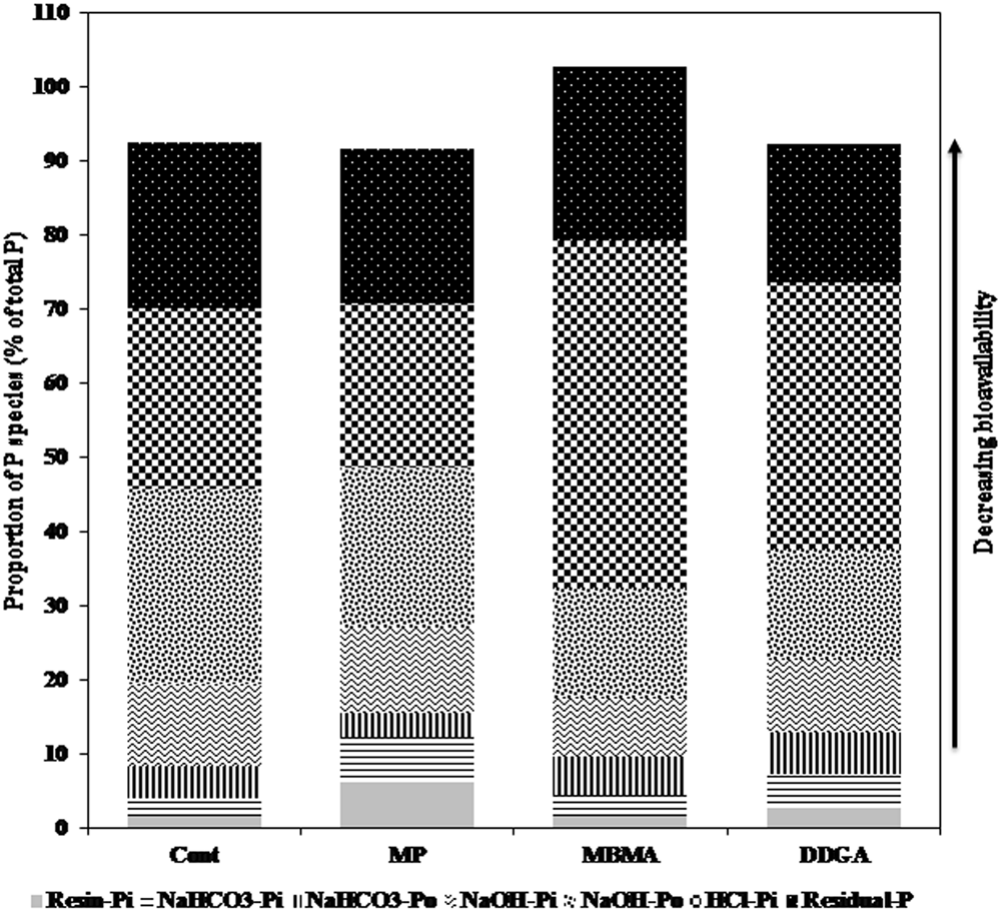
Proportions of P species (% of total soil P) in soil treated with mineral fertilizer (MP), meat and bone meal ash (MBMA) and dried distillers grains ash (DDGA) and untreated soil (Cont). The values are the mean of 3 rates of application for each P source treatment.

The concentration of inorganic P recovered from soil by anion exchange resin membrane strip, which is defined as freely exchangeable P_i_, was significantly affected by P source, rate of P application and their interaction (Table 3). The high rate of MP and DDGA treatments had the highest content of resin-P_i_, and both treatments were significantly higher than the control. Similarly, P source, rate of application and their interaction had a strong impact on concentration of NaHCO_3_-P_i_, showing a similar pattern of treatment effect to the resin fraction. The amount of organic P in NaHCO_3_extraction was significantly influenced by P source and its interaction with rate of application (Table 3). The greatest content of NaHCO_3_-P_o_ was observed with DDGA when applied at the high; this was significantly higher than the control and MP when applied at any rate (Table 3), but did not differ from that in MBMA treatments. When averaged over the 3 rates of application, the organic P concentration in NaHCO_3_ fraction was the highest in DDGA treatment, followed by MBMA and then MP treatments (Table 4). It accounted for 6%, 5% and 3% of total P in DDGA, MBMA and MP, respectively (Fig. 1). As expected, the labile P fractions of resin-P_i_ and NaHCO_3_-P (inorganic and organic) represented only a small proportion of the total P in the soils, ranging from 8%, in unamended soil, to 15% in MP treatments, averaged across the 3 rates of application (Fig. 1). This is consistent with previous studies conducted with grassland or cultivated Chernozemic soils in which the fraction of P recovered by resin and NaHCO_3_ did not exceed 10% of the total P^39,40^. In the MP treatments, the P content in resin fraction was consistently larger than both the MBMA and DDGA treatments (Table 2), and the same was also shown with NaHCO_3_-P_i_ fraction when values were averaged over the rates of application (Table 4). This can be explained by the P contained in MP in water soluble form. The labile P was higher in high rate DDGA treatment than that in MBMA, and this is consistent with the higher P uptake by the canola observed in the DDGA-amended soil^26^. A similar pot study examining P fractions in soil fertilized with poultry litter ash found that resin-P_i_ content in ash treated soil was higher than the control and comparable to that in soil treated with mineral P, suggesting high release of P from ash^16^. Other studies examining the potential use of ash as a P fertilizer found increases in soluble P after ash application. For instance, available P extracted with NaHCO_3_ increased in soil treated with gasified alfalfa stems^41^. Water soluble P was also found to be higher in soil treated with poultry litter^7^. In a pot experiment, addition of ash generated from straw gasification showed a significant increase in soil content of soluble P extracted with NaHCO_3_, whereas ash generated from citrus peel fiber gasification did not^42^. As also demonstrated in the current study, the level of P solubility in soil following ash application is mainly influenced by the rate of application as well as the P solubility in the ashes, which is variable according to feedstock and combustion/gasification processes. For instance, agricultural residue-derived ashes appear to exhibit greater P solubility than that in wooden biomass-derived ashes^43–46^ whereas ash generated from meat and bone meal mainly consisted of calcium phosphates (e.g. apatite), which are low in solubility^47,48^. Based on the above evidence, the variation in labile P in the current study may arise from distinct differences in the chemical composition of the ashes.

**Table 3:** Effects of meat & bone meal ash (MBMA) and dried distillers grain ash (DDGA) application on sequentially extracted P fractions in soil (mean ± SE).

Treatment		Resin P_i_	NaHCO_3_ P_i_	NaHCO_3_ P_o_	NaOH P_i_	NaOH P_o_	HCl P_i_	Residual P
P source	Rate (kg ha^−1^)	μg P g^−1^ soil						
Control	0	7 ± 4de	12 ± 5c	20 ± 4bc	52 ± 1abc	124 ± 11a	115 ± 10de	105 ± 8b
MP	25	15 ± 5 cd	32 ± 3ab	15 ± 1c	56 ± 2abc	116 ± 5a	70 ± 18e	103 ± 6bc
	50	22 ± 3bc	24 ± 2abc	17 ± 2c	52 ± 3abc	94 ± 7bc	119 ± 19de	105 ± 4bc
	100	54 ± 4a	32 ± 4ab	16 ± 1c	59 ± 2ab	100 ± 3b	132 ± 17 cd	96 ± 8bcd
MBMA	25	9 ± 2de	9 ± 3c	27 ± 3ab	39 ± 2bcd	69 ± 4d	198 ± 23b	107 ± 1b
	50	7 ± 2de	13 ± 3c	25 ± 1abc	39 ± 2bcd	76 ± 5 cd	179 ± 19bc	109 ± 4b
	100	8 ± 1de	20 ± 4bc	23 ± 1abc	38 ± 2 cd	73 ± 3 cd	313 ± 18a	129 ± 7a
DDGA	25	6 ± 1e	13 ± 1c	23 ± 1abc	32 ± 1d	65 ± 4d	158 ± 17bcd	90 ± 2 cd
	50	10 ± 1de	18 ± 2bc	29 ± 3ab	48 ± 10abcd	75 ± 3 cd	161 ± 7bcd	85 ± 1d
	100	25 ± 1b	35 ± 5a	31 ± 3a	62 ± 9a	76 ± 4 cd	199 ± 28b	93 ± 8bcd
		*P* > *F*						
Source of variation		***						
P source		***	***	***	***	***	***	**
Rate		***	***	ns	*	ns	***	ns
P source × Rate			*	*	*	**	*	ns

**Table 4:** Sequentially extracted P forms in soil treated with mineral P (MP), meat & bone meal ash (MBMA) or dried distillers grain ash (DDGA), averaged over rates of P application).

P Source	Resin P_i_	NaHCO_3_ P_i_	NaHCO_3_ P_o_	NaOH P_i_	NaOH P_o_	HCl P_i_	Residual P
				μg P g^−1^ soil			
MP	30a	29a	16b	56a	104a	107c	102b
MBMA	8b	15c	25a	39c	73b	230a	114a
DDGA	13b	22b	28a	47b	72b	173b	90c

The NaOH-extractable P fraction (P_i_ and P_o_) is considered moderately labile P^10^ and assumed to have low availability to plants^10,49^. The impact of rate of application on NaOH inorganic P fraction was more evident with DDGA treatment. In NaOH fraction, both inorganic and organic P were higher than the most labile P (resin-P_i_, NaHCO_3_-P_i_, NaHCO_3_-P_o_), with the NaOH-P_o_ representing 64% of total P (inorganic + organic) in this fraction, on average (Fig. 1). The soil content of inorganic and organic P in NaOH fraction varied amongst the various treatments, with the greatest amount observed with the control (Table 3). Generally, the percentage of P in NaOH fraction (P_i_ and P_o_) observed in this study is within the range of that found in different soil orders worldwide as reviewed by Cross and Schlesinger, 1995^49^. The addition of both ashes did not enrich the NaOH-P pool compared to the control, which is consistent with a previous study that reported that poultry litter ash addition did not significantly increase NaOH-P fraction when compared to the control^16^. It was also reported in a recent study that bone char had no significant impact on soil concentration of inorganic and organic forms of P extracted with NaOH^50^. In contrast, it has been documented in other studies that mineral fertilizer application resulted in an increase of NaOH-P fraction in soil^16,51^.

Phosphorus source, rate and their interaction all had a strong, significant impact on inorganic P content in HCl fraction (Table 3). On average, HCl-P_i_ pool was the dominant P pool in DDGA and MBMA treatments (Table 3). This fraction comprised 47%, 36% and 22% of the total P in MBMA, DDGA and MP treatments, respectively (Fig. 1). Phosphorus extracted with HCl constitutes more stable Ca-bound P in soils and is considered to have low plant availability^11,52^. Crystalline calcium phosphate (as apatite) is the major P-bearing component of bones and MBMA^47,48,53^. This explains not only the high concentration of P in HCl-P_i_ fraction in MBMA, but also the low crop availability of P in this ash type, as demonstrated earlier^26^. The MBMA may be of higher value if applied to acidic soil in which its P dissolution and release rate is expected to increase. In contrast with MBMA, the lower content of HCl-P_i_ in DDGA may be attributed to its lower content of Ca (8%), which likely resulted in less Ca-bound P in soil after application. This is consistent with the high abundance of available P fractions (resin-P_i_, NaHCO_3_-P_i_,) in high application rate of DDGA compared to MBMA. Furthermore, P in DDGA was very accessible by crop, similar to that in MP^26^.

The residual P fraction is regarded as having the lowest solubility and bioavailability^49^. This fraction was significantly influenced by P source, but not by rate or its interaction with P source (Table 3). The greatest amount of residual P was observed with MBMA, representing 24% of the total P present in this treatment in amended soils (Fig. 1).

### XANES analysis

**Figure 2:**
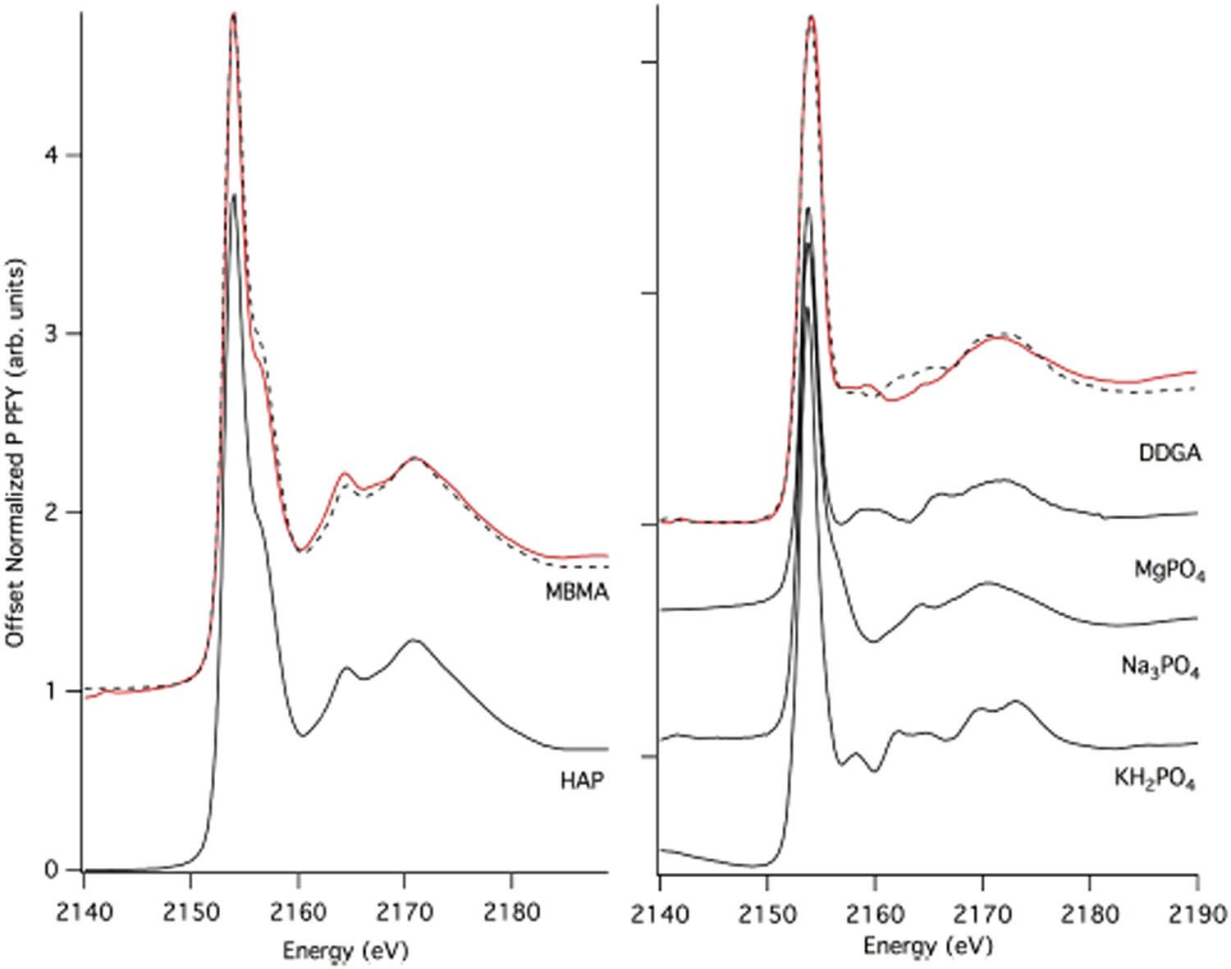
Comparison of MBMA and DDGA materials with reference compounds. Normalized data is in red and fits to compounds are denoted by the dashed line. (left) MBMA modeled as 100% hydroxylapatite (HAP). (right) DDGA modeled as a combination of 43% bobierrite (Mg phosphate), 23% Na_3_PO_4_, and 36% KH_2_PO_4_ salts.

Figure 2 compares P XANES spectra of the meat and bone meal ash (MBMA) and dried distiller’s grain ash fertilizers (in red) (DDGA) to reference compounds (hydroxyapatite, magnesium phosphate, and potassium phosphate) with similar spectral features. The MBMA ash exhibits strong spectral features consistent with hydroxyapatite, Ca_5_(PO_4_)_3_(OH), which is not considered plant available^47^. This is consistent with the large amount of calcium (24.65%) that MBMA contains (Table 1). In contrast, the DDGA ash weakly exhibits some spectral features which are consistent with a mixture of Mg, K and Na phosphate salts rather than a single mineral species. This is plausible as DDGA contains higher amount of Mg and K compared to MBMA (Table 1).

**Figure 3:**
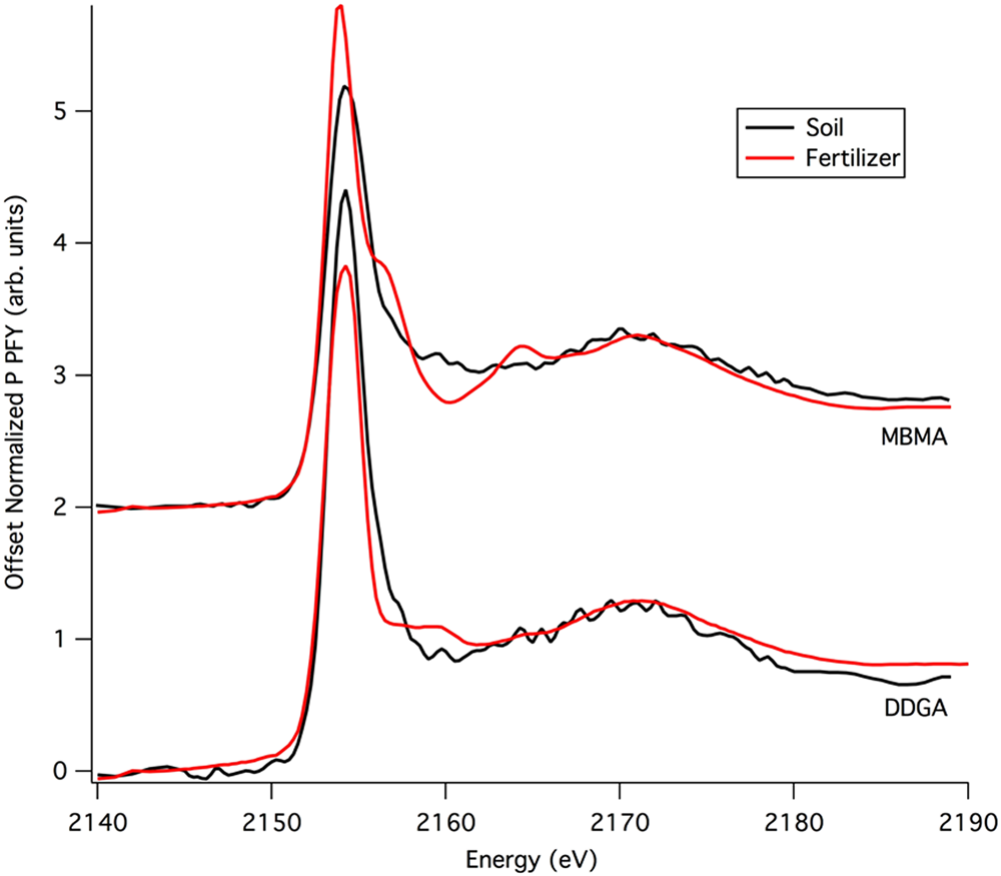
Comparison of the ash P XANES spectra prior to addition (red) with the P XANES spectra of the residual soil P after amendment and five weeks of canola crop growth. Both soil samples were from the 100 kg/ha P addition rate.

**Figure 4:**
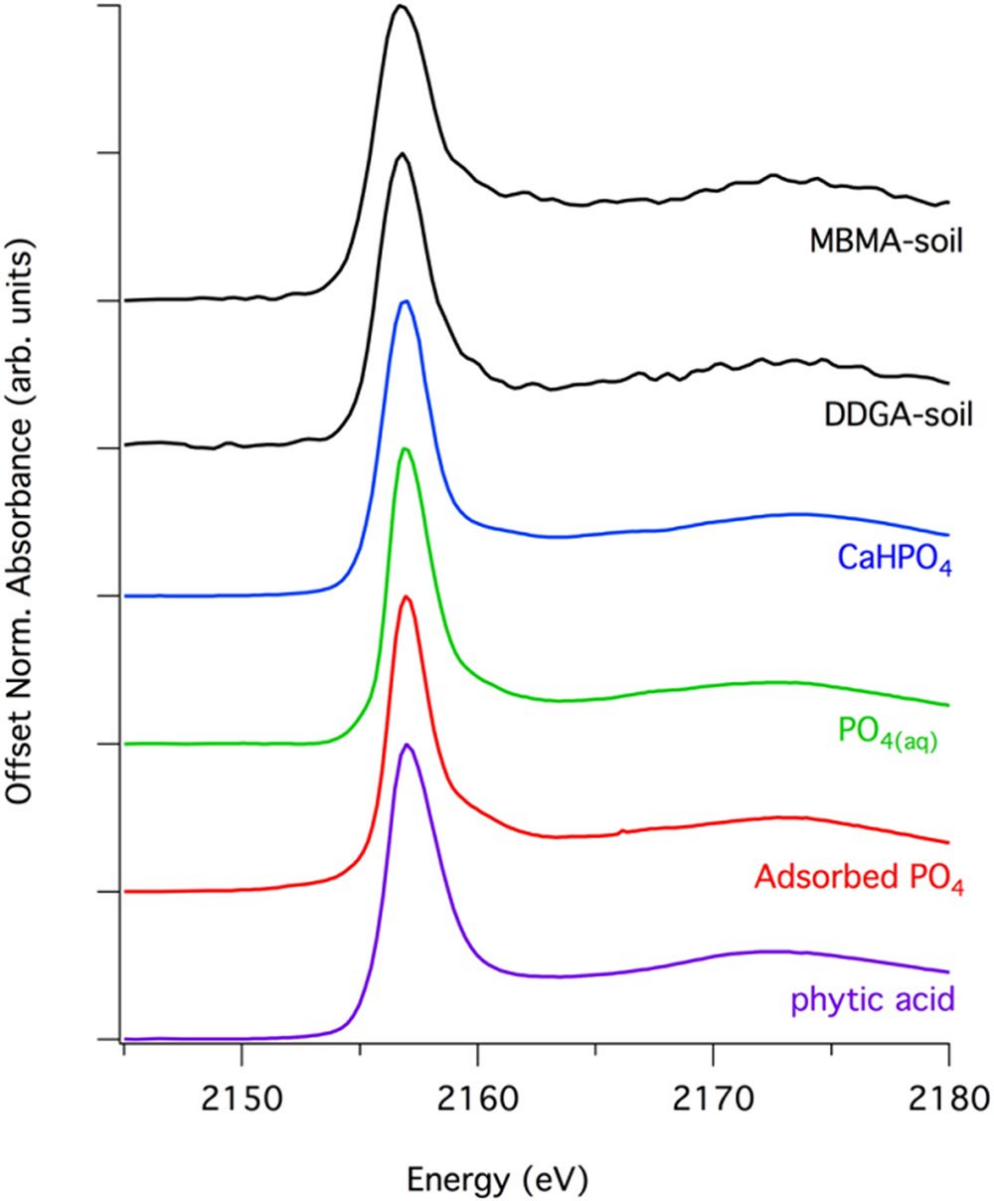
Comparison of the soil samples to a range of phosphate standards. Because there are no unique spectral fingerprints in the soil spectra, there is also no unique solution to linear combination XANES analysis for the samples.

Interestingly, all the distinctive features in the XANES spectra of both MBMA and DDGA fertilizers disappear after soil amendment and canola production; no obvious distinct features are observed (Fig. 3). This suggests that P in both ash fertilizers is transformed in the soil after application. The XANES data of the fertilized soil samples were also compared to a variety of phosphate standards (Fig. 4). The similarity of the MBMA and DDGA fertilized soil samples suggests that similar type of P transformation occurred after application regardless of source. It is also possible that the added amount of P to the studied soil via DDGS or MBMA was small enough so that P speciation is not different from the background P level. By assuming that the sum of the extracted P species in the studied soil is almost the total P, it appeared that there was no a huge increase in total P in treated soil. For the DDGA treatment applied at the high rate, the total P increased by 16%, which may indeed be difficult to identify with spectroscopy. However, there was a 39% increase in total P from the control to MBMA applied at the high rate treatment, and almost all this increase would be expected to be due to apatite-like phosphate which has a distinctive XANES spectra. This was not observed in the final samples, which supports our contention that the MBMA has undergone transformation during the trial. The lack of clear spectral features has been observed for a range of P standard materials including adsorbed phosphate, aqueous phosphate, amorphous calcium phosphate, and organic phosphate forms^20,24,25^. The absence of diagnostic spectral features of P forms present in the reference materials in Fig. 2 makes it difficult to perform linear combination fitting or quantitative peak identification in these soil samples because it is not possible to distinguish species with low spectral content (e.g. adsorbed, organic, amorphous phases, and dissolved phosphate) from one another. In these samples, any redistribution among adsorbed, aqueous, organic, and poorly crystalline Ca-P forms may not easily be tracked using XANES spectroscopy. Nonetheless, it is clear from our XANES measurements that P has transformed from the initial ash sources into a different, much less crystalline form when added to the soil and canola plants were grown on the soil for five weeks.

## Conclusion

The speciation of P in both ashes (MBMA and DDGA), and in the ash amended soils after 5 weeks of canola growth, demonstrated the transformation of ash P into different forms after application to soil. According to sequential extraction procedure, DDGA resulted in more soluble P species in soil compared to MBMA. In contrast, the MBMA treatment showed greater content of insoluble and recalcitrant species (HCl-Pi and residual-P), together representing approximately 70% of total soil P, averaged across the 3 rates of application. According to the HCl extractable P in the sequential extraction, more Ca-bound P is present and formed in soil receiving MBMA ash application, consistent with Ca and P contributed by bones. Analysis of MBMA with XANES spectroscopy before application to soil showed strong spectral features consistent with hydroxyapatite (Ca_5_(PO_4_)_3_(OH)), whereas DDGA weakly exhibits spectral features consistent with a mixture of several Mg and K phosphate salts rather than a single mineral species. The disappearance of distinctive features in the XANES spectra of both MBMA and DDGA after addition to soil is a possible indication that a transformation of ash P forms has occurred. It is also possible that the added amount of P to the studied soil via DDGS or MBMA was small enough so that P speciation is not different from the background P level. Overall, this study indicates that the nature of feedstock affects the chemical composition and P forms found in gasification ashes, which in turn affects the extractability of soil P after amendment. Transformation from initial P form is evident in spectroscopic assessments made after plant growth. Of the three P sources evaluated, based on the chemical and spectroscopic analysis the MBMA would appear to pose the lowest risk for P transport in water but is also the least effective as a rapid source of plant available P. However, MBMA may be of higher value if used in acidic soils where the rate of P dissolution and release is anticipated to increase. Future studies may consider transformations over longer terms (months, years) and a wide range of ashes varying in chemical composition applied to soils with contrasting properties, using a combination of P fractionation techniques, such as sequential extraction and other spectroscopic techniques, including XANES, NMR and FTIR.
